# Comparison of music and vapocoolant spray in reducing the pain of venous cannulation in children age 6-12: a randomized clinical trial

**DOI:** 10.1186/s12887-022-03271-9

**Published:** 2022-04-29

**Authors:** Masoomeh Ghasemi, Poria Hoseinialiabadi, Fatemeh Yazdanpanah, Maryam Askaryzadeh Mahani, Leila Malekyan, Kazem Najafi, Mohammadreza Arab, Mansour Arab, Hadi Ranjbar

**Affiliations:** 1 Medicine School, Bam University of Medical Sciences, Bam, Iran; 2 Student Research Committee of School of Medicine, Bam University of Medical Sciences, Bam, Iran; 3 Department of Nursing, School of Nursing and Midwifery, Bam University of Medical Sciences, Bam, Iran; 4 Department of Medical-Surgical Nursing, School of Nursing and Midwifery, Bam University of Medical Sciences, Bam, Iran; 5 Kerman University of Medical Sciences, Kerman, Iran; 6 Mental Health Research Center, Psychosocial Health Research Institute, Iran University of Medical Sciences, Tehran, Iran

**Keywords:** Pain, Venous cannulation, Music therapy, Vapocoolant spray, Children, Crossover trial

## Abstract

**Background:**

Venous cannulation is among the most stressful and painful experiences of children hospitalization. Children with thalassemia need regular blood transfusion which needs venous access each time. The quality of care and quality of life of children will be improved if appropriate methods are used to reduce pain. This study aimed to compare vapocoolant spray and music in the reduction of pain of Venous cannulation in children with thalassemia.

**Methods:**

The study was a randomized controlled clinical trial with a cross-over design. Thirty-six children with thalassemia from Thalassemia Patients of Pasteur Hospital in Bam from October to December 2020 and were recruited and randomly allocated to two arms. The pain of venous cannulation (no treatment) was measured in the first blood transfusion session as control. In the second and third sessions, two arms received music and vapocoolant spray before the venous cannulation with a cross-over design. The intensity of pain was measured by a Visual Analogue Scale (VAS). The change in pain scores was tested by ANOVA and Tukey post-hoc test between three measurements.

**Results:**

During and after the cannulation, the pain was significantly lower in the vapocoolant measurement than in control and music (*p* < 0.05). There was a significant effect of vapocoolant spray during the procedure *F* (2, 90) = 25.604, *p* = 0.001. Also, there was a significant effect of vapocoolant spray after the procedure *F* (2, 90) = 10.087, *p* = 0.004). Music did not reduce the pain during cannulation (*p* = 0.413) and after that (*p* = 0.807) significantly when compared with control.

**Conclusions:**

Vapocoolant was an effective method of pain reduction in the reduction of venous cannulation pain. Music was not effective in the reduction of venous cannulation pain when we compared it with controls. The pain of venous cannulation is rated as high and it can have negative effects on the children. There is a need to do more research on the methods of pain reduction of venous cannulation.

**Trial registration:**

The trial is registered: IRCT20111019007844N13, 13/03/2020. Available at: https://en.irct.ir/trial/42904.

## Introduction

Venous cannulation is one of the most common and painful invasive procedures in hospitalized children [1, 2]. Venous cannulation is ranked as the second source of severe pain in hospitalized children and is known as the most important cause of stress among them [3]. It is described by children as one of the most painful and frightening medical procedures [4]. Many efforts have been made to reduce the pain of this procedure. Several studies have been performed to find effective ways to reduce its pain [5–8]. However, the results of a systematic review showed that there is no consensus on the best method of pain reduction in this procedure [9].

Children with thalassemia need regular blood transfusion which needs venous access each time [10]. Venous cannulation is the most stressful aspect of treatment for patients with thalassemia. The pain of venous cannulation was reported by 39% of children with thalassemia very severe and by 42% as severe [11]. Long-term exposure to pain has devastating effects, including depression and PTSD, that should be avoided. The results of previous studies showed that mental health problems such as depression and anxiety are more prevalent in children with thalassemia [10]. Some of these problems are related to the treatment process and its complications like the pain of venous cannulation.

The reduction of pain of venous cannulation was the concern of researchers in the field of pain for several years. Using local anesthesia was among the first methods [1, 2]. Initially, injection of anesthetic solution at the venipuncture site with a thinner needle was considered. This method was very effective, but it required skill and an extra injection while increasing the risk of the needle stick [12]. The next method was to use Eutectic Mixture of Local Anesthetics (EMLA). This method is very effective but the need for time to effect, the possibility of skin allergies, and its cost limit its application [13, 14]. Newer methods developed during past years and the efforts continue. In particular, several studies have been performed on the pain of children due to its negative effects on the experience of hospitalization [15, 16]. However, the most effective method of relieving venipuncture pain has not yet been identified.

Knowledge about pain in children in the past two decades has increased significantly. Due to the side effects and limitations of pharmacological methods, non-pharmacological methods of pain reduction have been developed in the past two decades [17]. Distraction methods are of the most used methods of pain reduction which are studied before. They are practical, simple, and inexpensive non-pharmacological methods of pain relief [18]. Using music is one of the inexpensive methods of distraction during venipuncture [19]. Two theories support the effectiveness of music in the reduction of pain which are the gate control theory [20] and broaden-and-build theory of positive emotions [21].

The gate control theory of pain emphasizes that large myelinated fibers (Aβ fibers) that are associated with touch, pressure, and vibration, but not pain, can activate interneurons that modulate, or gate, firing of secondary spinal neurons associated with nociceptors (primary afferent pain fibers) [22]. According to the gate control theory, anxiety and fear open the gate and increase pain while music can calm the child and close the gate [22]. Based on the Broaden-and-Build Model, pain and anxiety are reduced when positive emotions are elicited by pleasant simulators like music and they can reduce the procedural pain [23]. Based on these models we expected that music can reduce the pain of venous cannulation.

Several studies showed that music is effective in the reduction of venous cannulation pain [24, 25]. Studies showed that vapocoolant sprays were effective methods in the reduction of pain. We did not find any study that compared the vapocoolant spray and music in the reduction of venous cannulation in children. Also, there is no consensus about the best method of pain reduction during venous cannulation. Music and vapocoolant spray are two inexpensive methods, that are easy to use and can be used in any setting. This study aimed to compare vapocoolant spray and music in the reduction of pain of Venous cannulation in children with thalassemia.

## Methods

### Design

This was a randomized clinical trial with a cross-over design. The study aimed to compare the effect of music and vapocoolant spray in the reduction of pain of venous cannulation. Thirty-six children with thalassemia were recruited. In the first blood transfusion, their pain was measured as control and then they were assigned randomly into 2 arms. The primary outcome was the pain experienced during the venous cannulation which was measured using a Visual Analogue Scale. We followed the CONSORT guidelines for reporting randomized controlled trials.

### Setting and sample

The study samples were selected from children with thalassemia who attended to Thalassemia Center in Bam, Iran for blood transfusion, from October to December 2020. They needed several blood transfusions with at least 2 weeks’ intervals. The inclusion criteria were age 6-12 years, being conscious, the ability to communicate in Persian, and the ability to respond to the visual scale of pain. Exclusion criteria were inability to respond to pain assessment scales, use of pain reliefs before or during the procedure, and not having consent to participate in the study. The minimum sample size was calculated using the following formula $\mathrm{n}=\frac{2{\left({\mathrm{Z}}_{\left(1-\upalpha /2\right)}+{\mathrm{Z}}_{1-\upbeta}\right)}^2{\upsigma}^2}{{\mathrm{d}}^2}$ and with the following: 1-α/2 = 1.96, 1-β = 1.64, σ = 31.9, and d = 30, and the calculated minimum sample size was 30. With a 20% drop-out rate, 36 children were recruited in the study. The convenience sampling method was used. Children were randomly assigned to one of the two arms, with a blocking design. Six blocks were defined (ABC, ACB, BAC, BCA, CAB, and CBA) and a number between 1 and 6 were assigned to each block. By rolling dice, the sequence of blocks was determined. Children with even numbers were allocated to arm A and Children with odd numbers were allocated to arm B. The first author and the statistician were not present in the patient enrollment and assignment (allocation was concealed), and the corresponding author was not involved in data analysis. Due to the nature of the intervention blinding was not possible. Cannulation was conducted by the same nurse for all subjects and pain measurement was conducted by two members of the research team who were not present at blocking concealment, and analysis.

### Data collection and processing

We used a cross-over design and all samples received both interventions along with a control measurement. The first author explained the purpose of the research and procedures to children and their parents. Parents and children were informed that venous cannulation will be based on the center routine. Also, the pain relief methods would only be applied with their consent. Children and their parents also received a full description of the used pain relief methods and how levels of pain would be measured. After full disclosure to children and their parents about sampling, pain relief methods, and measurement, written informed consent was obtained. For all children, the same size of catheters (22-gauge) made by the same producer was used. All cannulations were conducted by the same trained nurse with the same procedure in the brachial vein. The nurse who conducted the venous cannulation was working in the center and she was not a member of the research team.

**Fig. 1:**
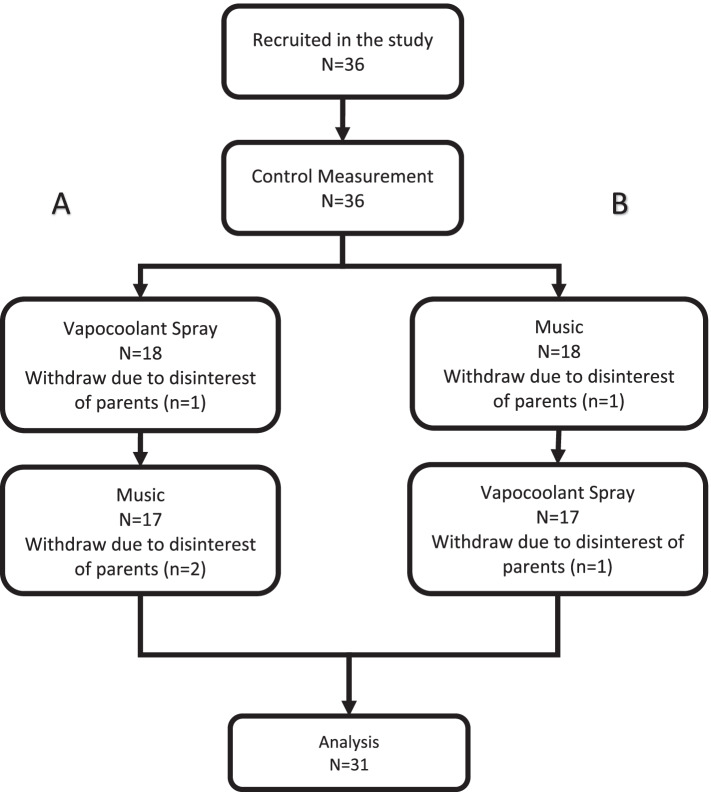
Study flowchart: recruitment and allocation to study arms

Because there was no routine method of pain relief in the center at the time of sampling, the first venous cannulation was conducted without any treatment and its pain was measured as the control. After control measurement study subjects were randomly assigned to two arms that received both interventions with different sequences within 4 weeks. The allocation and intervention processes are presented in Fig. 1. In the recruitment 36 children entered the study and control measurement was conducted for them. In the next transfusion session children in two arms (A and B) received the study intervention. In both arms one child was withdrawn due to the disinterest of parents, then both arms had 17 subjects in the second measurement. Arm (A) lost 2 children and arm (B) lost 1 child in the third session. In the end, 31 children completed all three measurements and their data was analyzed with ANOVA.

Subjects in arm A, received vapocoolant spray and music before the venous cannulation in the second and third blood transfusion, respectively. Subjects in arm B received the opposite sequence. Vapocoolant spray was applied from a 25 cm distance for 5 s and the cannulation was performed instantly [3]. Music intervention was conducted a follows: they listened to the music of choice of children before the cannulation through the headphones. Pieces of music were played for 30 min before the cannulation and the procedure was performed immediately.

### Pain measurement

The pain was the primary outcome of the study. The pain of venous cannulation was assessed using a Visual Analogue Scale (VAS). VAS is a validated scale whose usefulness in the measurement of the pain of pediatric patients is confirmed. It has one item which is scored from 0 to 6. Higher scores indicate a higher level of pain. The scale has good reliability and it was used in several studies [3]. Two raters separately observed each subject during and after the cannulation. The interrater reliability was high (*r* = 0.87). The mean of two scores is reported as the score of VAS.

### Statistical analysis

The data was entered into SPSS Version 16. The pain scores were reported as mean ± SD. The Shapiro-Wilk test was used to test for normality of VAS scores (*p* > 0.05). The change in pain scores was tested by ANOVA and Tukey post-hoc test between three measurements. The level of significance was set at *p* < 0.05 in all tests.

## Results

At the end of the study 31 subjects completed all three measurements. The mean ± standard deviation of the study subject’s age was 9.90 ± 1.86 years. The socio-demographic characteristics of study subjects are presented in Table 1.

**Table 1:** The socio-demographic characteristics of study subjects

Variable	N	Percentage
Gender		
Male	13	41.9
Female	18	58.1
School Grade		
First grade	5	16.1
Second grade	4	12.9
Third grade	6	19.4
Fourth grade	5	16.1
Fifth grade	9	29
Sixth grade	2	6.5
Father’s job		
Employed	28	87.1
Unemployed	3	9.7
Mother’s job		
Housekeeper	30	96.8
Government Employed	1	3.2
Education of father		
Illiterate	13	41.9
Below diploma and below	14	45.2
Diploma	4	12.9
Education of father		
Illiterate	11	35.5
Below diploma and below	16	51.6
Diploma	4	12.9
Place of residence		
Urban	10	32.3
Rural	21	67.7

During and after the cannulation, the pain was significantly lower in the vapocoolant measurement than in control and music (*p* < 0.05). A one-way ANOVA was conducted to determine that is there a statistically significant difference between vapocoolant spray, music, and control measurement on reported pain. There was a significant effect of vapocoolant spray during the procedure *F* (2, 90) = 25.604, *p* = 0.001. Also, there was a significant effect of vapocoolant spray after the procedure *F* (2, 90) = 10.087, *p* = 0.004). Music did not reduce the pain during cannulation (*p* = 0.413) and after that (*p* = 0.807) significantly when compared with control (Table 2).

**Table 2:** Comparison of the effect of vapocoolant spray, music with no intervention, during and after the venous cannulation

Phase	Measurement	Mean	SD	Tukey			ANCOVA
				Control	Vapocoolant Spray	Music	
During procedure	Control	4.41	0.71		0.001	0.413	*F* (2, 90) = 16.00, *p* = 0.001
	Vapocoolant Spray	4.19	0.65	0.001		0.001	
	Music	3.22	0.71	0.413	0.001		
After procedure	Control	2.25	57		0.001	0.807	*F* (2, 90) = 5.73, *p* = 0.004
	Vapocoolant Spray	2.16	0.63	0.001		0.001	
	Music	1.61	0.61	0.807	0.001		

**Fig. 2:**
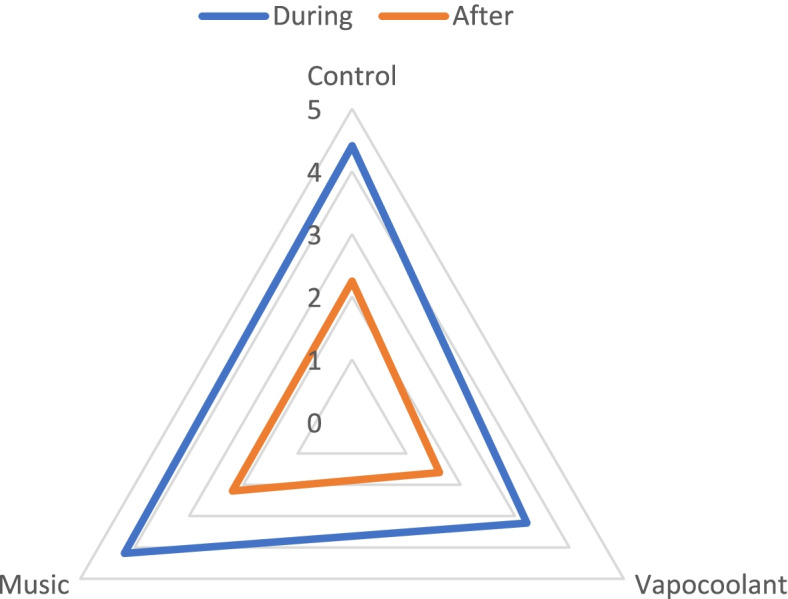
The changes in VAS scores in three methods: during and after venous cannulation. Figure 2 shows that the pain during cannulation (Blue triangle) was very high in control and music measurements. The Pain after cannulation (Orange triangle) decreased more significantly in the vapocoolant measurement

Figure 2 shows that in all three methods, a high degree of pain was reported during the procedure. The pain after using vapocoolant spray decreased more than music and control during and after the cannulation.

## Discussion

This study aimed to compare the effect of music and vapocoolant spray in the relief of venous cannulation pain in children aged 6-12 years. Our results showed that music did not have a significant effect on the reduction of pain, while vapocoolant spray had a significant effect. When we compared music with vapocoolant spry, the effect of vapocoolant spray was significantly higher.

The results of studies regarding the effect of vapocoolant sprays showed that they are moderately effective in the reduction of procedural pains [3, 26, 27]. When they were compared with methods with high power of pain reduction like using topical anesthesia (EMLA cream) they were not satisfactorily effective [28, 29]. But, when they were compared with controls, they were significantly effective in pain reduction during venous canulation. Vapocoolants are unstable liquids that act on the skin [3]. They can lower the temperature of the surface immediately by evaporating [30–32]. The quick cooling of the skin decreases the impulses speed that travels between nerve fibers [33]. They are efficient, inexpensive, and easy to apply methods of pain reduction [34]. While we found that they are effective methods of pain reduction in venous cannulation, the results of previous studies are not consistent. While some studies found them effective [26], the results of some studies found that they were not significantly effective in pain reduction [35, 36]. The results of a randomized clinical trial showed that vapocoolant spray was effective in the reduction of pain of venous cannulation but it was not as effective as EMLA [3]. A new meta-analysis found vapocoolant spray’s pain reduction effect significant in adults and children [37]. Our results confirmed the efficacy of vapocoolant spray on the reduction of pain of venous cannulation.

Our results showed that music intervention did not reduce the pain significantly when we compared it with the control measurement. The music was less effective than vapocoolant spray, as well. We did not find any study that compared the pain relief effect of music and vapocoolant spray. Music was less effective than bubble making [24] and using the riddle [25], while it was effective in comparison to controls. Some studies showed that it is not effective in the reduction of pain of procedural pain [38, 39]. The reason for this difference may be related to the method of delivering the music and the subjects. In our study children with thalassemia had a long history of venous cannulation which can affect their experience of pain. Music may have been more effective in situations where the pain is less predictable. We recommend further studies in this regard.

### Limitations

The main limitation of the current study was the small sample size which may cause lower generalizability of the results. The small sample size was due to the crossover design. With a sample size of 30, we can claim that vapocoolant spray has a significant effect that could reduce the pain by over 30 mm. Music was not as effective as vapocoolant spray. Larger sample size is needed to reject the effectiveness of music on the pain of venous cannulation. Sampling from children with thalassemia also reduces the generalizability to healthy children. Children with thalassemia have the experience of regular venous cannulation before the study which can affect their experience and pain reporting. Another limitation of this study was the lack of blindness. Because of the nature of interventions, blinding was not possible.

## Conclusions

The results of our study show that vapocoolant spray was effective in the reduction of venous cannulation pain, but the music was not effective. This study by examining the same patients across two different methods with a cross-over design contributes to our knowledge of which method leads to the highest pain reduction. We did not find any study that compared the vapocoolant spray, two inexpensive and easy-to-use methods, which can be used in different settings without complicated educations and equipment. Many children with thalassemia receive blood transfusion before age of 6 and they need also effective methods of pain reduction. We suggest using new methods of pain reduction for them and measuring their pain with FLACC. (Face, Legs, Activity, Cry, CONSOL ability). We also recommend to consider the pain method reductions to be assessed in multicenter RCTs with subjects above age of 12. Our results are the base of studies about pain reduction of music and vapocoolant spray in children aged 6 12 years.

## Data Availability

All data will be available on request. All requests should be sent to the corresponding author email and they will be available within 1 week.

## Abbreviations

EMLA: Eutectic Mixture of Local Anesthetics
VAS: Visual Analogue Scale
ANCOVA: Analysis of covariance

## References

[1] Shivashankar A, Nalini KB, Rath P (2018). The role of nonpharmacological methods in attenuation of pain due to peripheral venous cannulation: a randomized controlled study. Anesth Essays Res.

[2] Srivastava VK, Das PK, Gautam SK, Jaisawal P, Kadiyala VN, Rambhad S (2016). Comparative evaluation of volatile anaesthetic agents for attenuation of venous cannulation pain: a prospective, randomized controlled study. J Clin Diagn Res.

[3] Dalvandi A, Ranjbar H, Hatamizadeh M, Rahgoi A, Bernstein C (2017). Comparing the effectiveness of vapocoolant spray and lidocaine/procaine cream in reducing pain of intravenous cannulation: a randomized clinical trial. Am J Emerg Med.

[4] Atzori B, Hoffman HG, Vagnoli L, Patterson DR, Alhalabi W, Messeri A (2018). Virtual reality analgesia during venipuncture in pediatric patients with onco-hematological diseases. Front Psychol.

[5] Bourdier S, Khelif N, Velasquez M, Usclade A, Rochette E, Pereira B (2021). Cold vibration (buzzy) versus anesthetic patch (EMLA) for pain prevention during cannulation in children: a randomized trial. Pediatr Emerg Care.

[6] Aykanat Girgin B, Gol I (2020). Reducing pain and fear in children during venipuncture: a randomized controlled study. Pain Manag Nurs.

[7] Garcia-Aracil N, Ramos-Pichardo JD, Castejon-de la Encina ME, Jose-Alcaide L, Julia-Sanchis R, Sanjuan-Quiles A (2018). Effectiveness of non-pharmacological measures for reducing pain and fear in children during venipuncture in the emergency department: a vibrating cold devices versus distraction. Emergencias.

[8] Bagheriyan S, Borhani F, Abbaszadeh A, Ranjbar H. The effects of regular breathing exercise and making bubbles on the pain of catheter insertion in school age children. Iran J Nurs Midwifery Res. 2011;16(2):174–80 Epub 2012/01/10. PubMed PMID: 22224103; PubMed Central PMCID: PMCPMC3249769. PMC3249769 22224103

[9] Ranjbar A, Bernstein C, Shariat M, Ranjbar H. Comparison of facilitated tucking and oral dextrose in reducing the pain of heel stick in preterm infants: a randomized clinical trial. BMC Pediatr. 2020;20:162. 10.1186/s12887-020-2020-7. 10.1186/s12887-020-2020-7 PMC7155270 32290829

[10] Al-Hakeim HK, Najm AH, Al-Dujaili AH, Maes M (2020). Major depression in children with transfusion-dependent thalassemia is strongly associated with the combined effects of blood transfusion rate, Iron overload, and increased pro-inflammatory cytokines. Neurotox Res.

[11] Vosuoghi N, Chehrzad M, Muosavi S, Atrkarruoshan Z, Akbari A (2009). Survey the effect of distraction on average heart rate due to IV insertion in 3-6. J Holist Nurs Midwifery.

[12] Farshchian M, Kimyai-Asadi A, Daveluy S. Cryosnip for skin tag removal. J Am Acad Dermatol. 2021. 10.1016/j.jaad.2021.05.039 Epub 2021/06/02. PubMed PMID: 34062213. 10.1016/j.jaad.2021.05.039 34062213

[13] Matsumoto T, Chaki T, Hirata N, Yamakage M (2018). The eutectic mixture local anesthetics (EMLA) cream is more effective on venipuncture pain compared with lidocaine tape in the same patients. JA Clin Rep.

[14] Sari E, Bakar B (2018). Which is more effective for pain relief during fractionated carbon dioxide laser treatment: EMLA cream or forced cold air anesthesia?. J Cosmet Laser Ther.

[15] Naik VM, Mantha SSP, Rayani BK (2019). Vascular access in children. Indian J Anaesth.

[16] Borzych-Duzalka D, Shroff R, Ariceta G, Yap YC, Paglialonga F, Xu H (2019). Vascular access choice, complications, and outcomes in children on maintenance hemodialysis: findings from the International Pediatric Hemodialysis Network (IPHN) Registry. Am J Kidney Dis.

[17] Hsieh KH, Chen SJ, Tsao PC, Wang CC, Huang CF, Lin CM (2019). The analgesic effect of non-pharmacological interventions to reduce procedural pain in preterm neonates. Pediatr Neonatol.

[18] Gates M, Hartling L, Shulhan-Kilroy J, MacGregor T, Guitard S, Wingert A, et al. Digital technology distraction for acute pain in children: a meta-analysis. Pediatrics. 2020;145(2). 10.1542/peds.2019-1139 Epub 2020/01/24. PubMed PMID: 31969473. 10.1542/peds.2019-1139 31969473

[19] Tapar H, Karaman T, Dogru S, Karaman S, Suren M, Altıparmak F (2019). Evaluating the efficacy of Valsalva’s maneuver and music therapy on peripheral venous cannulation: a prospective study. Anaesth Pain Intensive Care.

[20] Rezai MS, Goudarzian AH, Jafari-Koulaee A, Bagheri-Nesami M (2017). The effect of distraction techniques on the pain of venipuncture in children: a systematic review. J Pediatr Rev.

[21] Arewasikporn A, Ehde DM, Alschuler KN, Turner AP, Jensen MP (2018). Positive factors, pain, and function in adults with multiple sclerosis. Rehabil Psychol.

[22] Pereira PJS, Lerner EA (2017). Gate control theory springs a leak. Neuron.

[23] Buche H, Michel A, Piccoli C, Blanc N (2021). Contemplating or acting? Which immersive modes should be favored in VR during physiotherapy for breast cancer rehabilitation. Front Psychol.

[24] Momen Nasab M, Safawi M, Fesharaki M (2020). Investigating the effect of two distraction methods on venipuncture induced pain in children in Hazrat Masumeh Subspecialty Hospital in Qom. Med Sci J.

[25] Hoseini T, Golaghaie F, Khosravi S (2019). Comparison of two distraction methods on venipuncture pain in children. J Arak Univ Med Sci.

[26] Mace SE (2017). Prospective, double blind, randomized, controlled trial comparing vapocoolant spray versus placebo spray in adults undergoing intravenous cannulation. Scand J Pain.

[27] Mace SE (2016). Prospective, randomized, double-blind controlled trial comparing vapocoolant spray vs placebo spray in adults undergoing venipuncture. Am J Emerg Med.

[28] Gupta NK, Upadhyay A, Dwivedi AK, Agarwal A, Jaiswal V, Singh A (2017). Randomized controlled trial of topical EMLA and vapocoolant spray for reducing pain during wDPT vaccination. World J Pediatr.

[29] Moon YE, Kim SH, Seok H, Lee SY (2020). Comparison of the effects of vapocoolant spray and topical anesthetic cream on pain during intraarticular injection of the shoulder: a randomized double-blind controlled trial. Arch Phys Med Rehabil.

[30] Walsh ME, Schade S, Mitchell BK (2018). Review of article: Rusch, D., Florian Seel, T.K., Eberhart, L. Vapocoolant spray versus lidocaine infiltration for radial artery cannulation: a prospective, randomized, controlled clinical trial. J Cardiothoracic and Vascular anesthesia 2017;31:77-83. J Vasc Nurs.

[31] Rusch D, Koch T, Seel F, Eberhart L (2017). Vapocoolant spray versus lidocaine infiltration for radial artery cannulation: a prospective, randomized, controlled clinical trial. J Cardiothorac Vasc Anesth.

[32] Richardson C, Ovens E (2016). Therapeutic opportunities when using vapocoolants for cannulation in children. Br J Nurs.

[33] Farahmand S, Mirfazaelian H, Sedaghat M, Arashpour A, Saeedi M, Bagheri-Hariri S (2017). Vapocoolant spray effectiveness on arterial puncture pain: a randomized controlled clinical trial. Acta Med Iran.

[34] Mlynek K, Lyahn H, Richards B, Schleicher W, Bassiri Gharb B, Procop G (2015). Skin sterility after application of a vapocoolant spray part 2. Aesthet Plast Surg.

[35] Griffith RJ, Jordan V, Herd D, Reed PW, Dalziel SR (2016). Vapocoolants (cold spray) for pain treatment during intravenous cannulation. Cochrane Database Syst Rev.

[36] Hogan ME, Smart S, Shah V, Taddio A (2014). A systematic review of vapocoolants for reducing pain from venipuncture and venous cannulation in children and adults. J Emerg Med.

[37] Zhu Y, Peng X, Wang S, Chen W, Liu C, Guo B (2018). Vapocoolant spray versus placebo spray/no treatment for reducing pain from intravenous cannulation: a meta-analysis of randomized controlled trials. Am J Emerg Med.

[38] Shiroshita Y, Muraki K, Kamei T, Sobue I (2018). Pain-relieving effect of music on preschoolers during immunization: a randomized controlled trial. Health.

[39] Reza N, Ali SM, Saeed K, Abul-Qasim A, Reza TH (2007). The impact of music on postoperative pain and anxiety following cesarean section. Middle East J Anesthesiol.

